# Art therapy and music reminiscence activity in the prevention of cognitive decline: study protocol for a randomized controlled trial

**DOI:** 10.1186/s13063-017-2080-7

**Published:** 2017-07-12

**Authors:** Rathi Mahendran, Iris Rawtaer, Johnson Fam, Jonathan Wong, Alan Prem Kumar, Mihir Gandhi, Kenny Xu Jing, Lei Feng, Ee Heok Kua

**Affiliations:** 1 Department of Psychological Medicine, Yong Loo Lin School of Medicine, National University of Singapore, Singapore, Republic of Singapore; 2 Department of Psychological Medicine, National University Hospital, Tower Block, Level 9, 1E Kent Ridge Road, Singapore, Republic of Singapore; 3 Duke-NUS Medical School, Singapore, Republic of Singapore; 4 Cancer Science Institute of Singapore, National University of Singapore, 14 Medical Drive, Singapore, Republic of Singapore; 5 Department of Pharmacology, Yong Loo Lin School of Medicine, National University of Singapore, 21 Lower Kent Ridge Road, Singapore, Republic of Singapore; 6 Curtin Health Innovation Research Institute, Biosciences Research Precinct, School of Biomedical Sciences, Faculty of Health Sciences, Curtin University, Kent Street, Bentley, Perth, Western Australia Australia; 7 Department of Biological Sciences, University of North Texas, 1511 W Sycamore, Denton, TX USA; 8 Department of Biostatistics, Singapore Clinical Research Institute, Singapore, Republic of Singapore; 9 Centre for Quantitative Medicine, Office of Clinical Sciences, Duke-NUS Medical School, Singapore, Republic of Singapore; 10 Tampere Center for Child Health Research, University of Tampere and Tampere University Hospital, Tampere, Finland

**Keywords:** Art therapy, Music reminiscence activity, Mild cognitive impairment, Randomized controlled trial

## Abstract

**Background:**

Attention has shifted to the use of non-pharmacological interventions to prevent cognitive decline as a preventive strategy, as well as for those at risk and those with mild cognitive impairment. Early introduction of psycho-social interventions can address cognitive decline and significantly impact quality of life and the wellbeing of elderly individuals. This pilot study explores the feasibility of using art therapy and music reminiscence activity to improve the cognition of community living elderly with mild cognitive impairment.

**Methods/Design:**

This open-label, interventional study involves a parallel randomized controlled trial design with three arms (two intervention arms and a control group) over a nine-month period. Participants will be community-living elderly individuals aged 60–85 years, both genders, who meet predefined inclusion and exclusion criteria. In the initial three months, interventions will be provided weekly and for the remaining six months fortnightly. A sample size of 90 participants is targeted based on expected neuropsychological test performance, a primary outcome measure, and drop-out rates. The randomization procedure will be carried out via a web-based randomization system. Interventions will be provided by trained staff with a control group not receiving any intervention but continuing life as usual. Assessments will be done at baseline, three months, and nine months, and include neuroimaging to measure cerebral changes and neuropsychological tests to measure for changes in cognition. Secondary outcome measures will include mood changes in anxiety and depression and telomere lengths. Statistical analysis will be undertaken by statisticians; all efficacy analysis will be carried out on an intention-to-treat basis. Primary and secondary outcomes will be modeled using the linear mixed model for repeated measurements and further analysis may be undertaken to adjust for potential confounders.

**Discussion:**

This will be the first study to compare the effectiveness of art therapy and music reminiscence activity in a randomized controlled trial. We expect that the trial will provide useful evidence for developing psychosocial interventions for the elderly with mild cognitive impairment.

**Trial registration:**

The study was registered on 7 July 2016 at Clinical Trials.gov, a service of the US National Institute of Health (NCT02854085), retrospectively.

**Electronic supplementary material:**

The online version of this article (doi:10.1186/s13063-017-2080-7) contains supplementary material, which is available to authorized users.

## Background

Population aging is a global public health concern and dementia is one of the major causes of disability and dependency in the elderly population worldwide [1]. Research now targets the prevention or delay of cognitive decline as a more beneficial approach and attention has shifted to non-pharmacological interventions and preventive strategies for cognitively normal elderly and those exhibiting mild cognitive problems.

Mild cognitive impairment (MCI, DSM-5 Minor Neurocognitive Disorder) [2] is associated with high burden with about 50% of individuals going on to develop dementia within five years. While 14–40% may return to normal cognitive functioning over time, others may exhibit a persistent form of MCI without conversion [3, 4]. Interventions from stimulating leisure activities and an active cognitive lifestyle to address cognitive decline at this stage, have a significant impact on the quality of life and wellbeing of elderly individuals and also illness progression [5]. Evidence suggests that the causes of aging are multifactorial, ranging from increased inflammation and oxidative stress to decreased mitochondrial function, hormonal levels, and genome instability [6]. However, the aging brain still exhibits plasticity which allows interventions and cognitive training to bring about alterations in cerebral morphology and function [7].

A systematic review of psycho-social interventions (69 prospective controlled trials) found positive effects on quality of life and positive mental health [8] and engaging in stimulating activities at least twice a week reduced the risk of dementia by 50% [9]. Interventions focusing mainly on lifestyle modifications, notably the FINGER trial, have reported significant benefits from a multifaceted intervention that included diet, exercise, cognitive training, and vascular monitoring [10].

Our earlier work in an open-label trial of art therapy (AT), music reminiscence, mindfulness practice, and Tai Chi exercise with community-living elderly, revealed improvements in sub-syndromal anxiety and depressive symptoms [11]. This was followed by a randomized controlled trial (RCT) to examine Mindfulness Awareness Practice (the control group received Health Education) in the elderly with MCI and designed with a nine-month duration deemed sufficient for intervention response [12]. Interventions were provided weekly for three months and then monthly for six months and a range of “psychobiomarkers” were investigated [13].

Telomere length was one of those measured as it is widely accepted as a biomarker for cellular aging and cognitive decline with changes from various activities resulting in longer telomeres [14–16]. Preliminary findings from the Mindfulness Awareness Practice RCT have revealed changes in functional brain activity, neuropsychological tests, telomere lengths, and oxidative stress markers after just three months of weekly intervention (manuscript in preparation). Importantly, the frequency of the activity had a significant impact on the sustainability of gains produced by the intervention [17]. Existing research on AT and music reminiscence activity (MRA), however, is limited, especially on the efficacy of these interventions on cognition. Kim has shown that AT improves mood and overall wellbeing of elderly individuals, but the cognitive status of the participants was unknown [18]. While Alders and Levine-Madori have results that seem to suggest that AT is useful in improving cognitive performance [19], the study methodology was not rigorous. The experimental group could choose the activity to participate in and could attend as few as three of the ten AT sessions. The study by Rusted et al. [20] did not clearly explain the control group activity and recruited a wide range of dementia participants. None of the studies have been of sufficient rigor or focused on elderly with MCI.

Listening to music effectively stimulates the auditory cortex and other brain areas related to attention, semantic processing, memory, motor function, and emotional processing [21]. Yet studies on the specific use of MRA and its efficacy for elderly with MCI are rare. Most of the research has examined music therapy, with components of reminiscence, for mood symptoms, where listening to music they preferred [22] and choosing the type of music to listen to reduced anxiety and depression in the elderly [23, 24]. Petrovsky et al. did a review of the utility of music interventions on mood in the elderly with mild dementia and found that many of the studies were not methodologically strong, indicating that the evidence was inconclusive [25].

The research on music therapy and cognitive changes however has shown positive improvements on Mini-Mental State Examination (MMSE) scores, even one month post intervention [24] and on the short-term for working memory and quality of life in elderly with mild or moderate dementia [26]. However, the efficacy of music therapy for cognitive improvement is still debated and there is a gap in the literature for music reminiscence activities in elderly with MCI.

## Methods/Design

### Aims of study

This pilot study aims to explore the feasibility of using AT and MRA to improve the cognition of community living elderly with MCI (Diagnostic and Statistical Manual-5: Mild Neurocognitive Disorder) [2].

The primary outcomes will be the structural cerebral changes that occur with the two interventions and the extent to which the therapies may reverse cognitive impairment and/or prevent further cognitive decline on neuropsychological tests of cognition. This will be based on participants’ pre-, three-month and nine-month neuropsychological test scores, compared with individuals in the control arm who do not participate in active interventions.

Secondary outcomes comprise changes in blood biomarkers, psychological wellbeing, and mood symptoms.

We hypothesize that participants in both active intervention arms will have (1) improved functional connectivity, (2) improved neuropsychological cognitive test scores, (3) increased telomere lengths, and (4) enhanced psychological wellbeing and reductions in anxiety and depressive symptoms compared with the control group.

No a priori hypotheses were developed as to whether AT or MRA is more effective as the comparison is exploratory.

### Study design

**Fig. 1:**
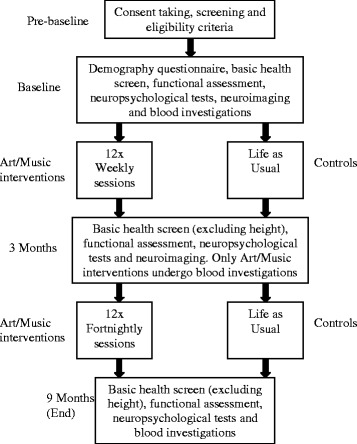
Workflow plan for participants

**Fig. 2:**
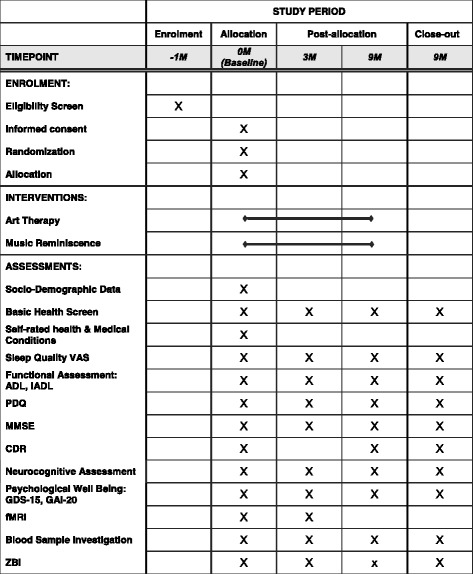
Standard Protocol Items: Recommendation for Interventional Trials (SPIRIT) figure: the schedule of enrolment, interventions, and assessments. *M* months, *VAS* visual analog scale, *ADL* Barthel Index (Activities of Daily Living), *iADL* instrumental Activities of Daily Living, *PDQ* Perceived Deficits Questionnaire, *MMSE* Mini-Mental State Examination, *CDR* Clinical Dementia Rating, *GDS-15* Geriatric Depression Scale (15-item), *GAI-20* Geriatric Anxiety Inventory, *fMRI* functional magnetic resonance imaging, *ZBI* Zarit Burden Inventory

This is an open-label, interventional study involving a parallel RCT design with three arms and run over nine months. The duration of nine months was used in previous RCTs of psychosocial interventions at our center and provided a trial length of sufficient duration which yielded measurable changes. The protocol was prepared according to the Standard Protocol Items: Recommendations for Interventional Trials (SPIRIT) guidelines (see Fig. 1, Additional file 1, and Fig. 2). Interventions will be weekly for the first three months, then fortnightly for the remaining six months. Weekly interventions provide greater practice effects and improvements in cognition and brain function, if any, can be detected by three months. In the subsequent six months, the frequency of the interventions has been increased to fortnightly, based on previous trial experience that monthly interventions do not sustain the early gains. Study evaluations will be done by comparing the two intervention groups (AT and MRA) and a control group. Participants will have written informed consent taken, screening, and baseline measures completed before randomization into one of the three study arms.

Intervention Arm 1: Participants will receive AT

Intervention Arm 2: Participants will receive MRA

Control Arm: Participants will not receive any intervention but continue their life as usual.

### Eligibility criteria

The inclusion criteria are:

1. Aged 60–85 years, both genders, and living in the community and fulfils the operational definition/criteria of MCI: (a) at least one age-education adjusted neuropsychological test Z score < −1.5; (b) do not meet DSM V criteria for a major neurocognitive disorder; (c) memory/cognitive complaint preferably corroborated by a reliable informant;
2. Functions independently as assessed with Barthel’s Activities of Daily Living and instrumental Activities of Daily Living;
3. Able to travel to the data collection site on their own and participate in the activity weekly for 12 weeks and then fortnightly for six months.

The exclusion criteria are:

1. Those who do not meet the above inclusion criteria (i.e. do not have MCI diagnosis);
2. Those with dementia/major neurocognitive disorder or normal aging;
3. Presence of a neurological condition, e.g. epilepsy, Parkinson’s disease, stroke;
4. Presence of a major psychiatric disorder, e.g. major depression, psychoses;
5. Terminal illness, e.g. cancer;
6. Presence of significant visual and/or hearing impairment and color blindness;
7. Participants in another intervention study at the same time.

### Randomization procedure

Participants will be randomized (on the same day after taking written informed consent, screening, and completing baseline measures) to the AT, MRA, or control groups at a 1:1:1 allocation ratio. Balanced intervention assignments will be achieved using permuted block randomization stratified by gender. Block size will be determined by the statistician responsible for generating the randomization list and will not be made known to the clinical investigator or site personnel.

Implementation of the randomization process will be carried out via a web-based randomization system by Singapore Clinical Research Institute. The randomization system will then determine the intervention arm and provide a unique participation number to be used for the participant.

### Delivery of interventions

The interventions will be delivered by trained art and music therapists. Activities in each intervention is structured and planned for the whole study period.

### Art therapy

The AT activity was developed by the team in consultation with art therapists. It will have two components: creation of art pieces; and viewing and discussing art pieces with narration of thoughts and inner experiences. Studies have shown that visual art production interventions and cognitive art evaluation groups benefit the individual but in slightly different ways [27]. Hence, this study will include both activities and consider them as one intervention.

The guided viewing and making of visual images and talk therapy with an art therapist contributes to externalization of thoughts and feelings which may otherwise remain unexpressed. Each weekly session will begin with mindful relaxation to help the participants focus on the task ahead. They will be asked to draw anything they want or what they think is relevant to the topic for each session. They will then be asked to share about the picture they have created, feelings, and perspectives, first in pairs and then with the group where image appreciation activities will be used to help participants gain insights and discuss their feelings.

In addition, participants will visit both the National Art Gallery and the National University of Singapore Art Museum once a month in the first three months. They will view displayed artworks in guided sessions and will be invited to share their views and feelings about the artefacts. Each of the AT sessions will last 1 h (inclusive of 5 min of mindful relaxation and a 15-min break). From the fourth to ninth month, fortnightly AT sessions are provided with sessions at the study center alternating with sessions at the National Art Gallery or National University of Singapore Art Museum. Participants will visit the Art Gallery and Art Museum, first, because the art pieces cannot be removed from these places and brought to the research center; second, two sites are involved because neither one of the sites has sufficient docents to manage the number of visits alone. With two sites, each site will host the participants’ visit only once a month in the first three months and then in alternate months for the next six months. Participants will be taken to the Art Gallery and Museum by coach and will be accompanied by research staff during the 15-min journey.

#### Content of the art therapy intervention

Weekly sessions:

- Session 1: Art and expression
- Session 2: Friendship
- Session 3: Visit to the NUS Art Museum
- Session 4: Visit to the National Art Gallery
- Session 5: Emotions and feelings
- Session 6: Family
- Session 7: Visit to the NUS Art Museum
- Session 8: Visit to the National Art Gallery
- Session 9: Happiness
- Session 10: Hopes and wishes
- Session 11: Visit to the NUS Art Museum
- Session 12: Visit to the National Art Gallery

Fortnightly sessions:

- Session 13: Art activity
- Session 14: Visit to the NUS Art Museum
- Session 15: Art activity
- Session 16: Visit to the National Art Gallery
- Session 17: Art activity
- Session 18: Visit to the NUS Art Museum
- Session 19: Art activity
- Session 20: Visit to the National Art Gallery
- Session 21: Art activity
- Session 22: Visit to the NUS Art Museum
- Session 23: Art activity
- Session 24: Visit to the National Art Gallery

### Music reminiscence activity

Reminiscence therapy with music entails listening and discussing activities, events, and experiences related to the music. Sessions will be presented as music videos, with additional prompts in the form of photographs, to facilitate therapy. Participants who agree will be asked to bring 10–20 photographs and also choose songs or music for the videos. Reminiscence provokes shared feelings and boosts self-esteem while the group process provides validation for each member [28]. The structured activity was previously planned by the team and used effectively in the earlier naturalistic study [11]. Participants will meet weekly for the first three months and then fortnightly for six months. As in the AT intervention group, each session will begin with a 5-min mindful relaxation and the 1-h session will include a 15-min break.

#### Content of the music reminiscence activity

Weekly sessions:

- Session 1: Introduction and ice-breaking session
- Session 2: Family of origin
- Session 3: House/Place I grew up in
- Session 4: Childhood
- Session 5: Childhood games
- Session 6: School
- Session 7: My favorite teacher, subject, classmate
- Session 8: Occupation
- Session 9: Significant events/achievements
- Session 10: Friendships
- Session 11: My best friend
- Session 12: Family/Marriage

Fortnightly sessions:

- Session 13: Parents/Children
- Session 14; Hobbies, sports and games
- Session 15: Holidays and travel
- Session 16: My favorite place in Singapore
- Session 17: Favorite food
- Session 18: Memorable songs
- Session 19: Festivals
- Session 20: Famous actors/singers
- Session 21: Recreational places
- Session 22: Old movies
- Session 23: Favorite parks
- Session 24: Termination; group discussion

### Control group

The control group will not receive any intervention but continue their life as usual.

### Study setting

Data collection will be done by trained nurses and research assistants blinded to the group assignment at a community research center used by the university, named the Training and Research Academy at Jurong Point (TaRA@JP).

### Measures

A questionnaire on demography will include a basic health screen (weight, height, BP, and PR at baseline and repeated [with the exception of height] at three and nine months). Functional assessment will be done using the Activities of Daily Living (ADL) questionnaire and the instrumental ADL (iADL).

Subjective cognitive impairment will be assessed using the Perceived Deficits Questionnaire (PDQ). It consists of 20 questions answered on a five-point Likert scale. Scores range from 0 to 80, with higher scores indicating severe impairment.

Cognitive assessments will be done with the following validated assessment scales:

1. Mini-Mental State Examination (MMSE), a brief 30-point questionnaire, will be used to screen for cognitive impairment. Scores range from 0 to 30, with higher scores indicating less impairment;
2. Clinical Dementia Rating (CDR) Scale is a five-point scale used to characterize six domains of cognitive and functional performance applicable to Alzheimer’s disease and other dementias: memory, orientation, judgment and problem-solving, community affairs, home and hobbies, and personal care;
3. Neuropsychological tests:
  - Rey Auditory Verbal Learning Test (RAVLT) evaluates declarative verbal learning and memory;
  - Digit Span Task consists of a Digit Span Forward (DSF) and Digit Span Backward (DSB) and is used to assess attention and verbal working memory;
  - Color Trails Test (CTT) 1 and 2 assess sustained attention and memory;
  - Block Design, a subtest of the Wechsler Intelligence Tests, measures visual–spatial and organizational processing abilities and non-verbal problem-solving skills.

Psychological wellbeing assessment will be assessed with the:

1. Geriatric Depression Scale (GDS): a 15-item “yes/no” questionnaire with higher total scores associated with higher risk of depression;
2. Geriatric Anxiety Inventory (GAI): a 20-item “agree/disagree” questionnaire measuring dimensional anxiety, with higher total scores associated with anxiety symptoms.

Participants with high GDS score (≥5) and/or high GAI score (≥ 10) will be clinically assessed using the Structured Clinical Interview for DSM Disorder (SCID) to rule out major psychiatric disorders (e.g. generalized anxiety disorder or major depressive disorder).

#### Neuroimaging assessment of functional connectivity and structure of the brain

Functional magnetic resonance imaging (fMRI) will be employed to examine changes in functional connectivity. Images will be acquired on a 3 T Siemens scanner using a standard Siemens whole head coil. Twenty-eight axial slices (4-mm thick, 1-mm skip) parallel to the plane connecting the anterior and posterior commissures and covering the whole brain will be imaged using a T2*-weighted gradient echo spiral pulse sequence (repetition time/echo time = 2000/30 ms; flip angle = 80°; and interleave = 1). The field of view will be 200 × 200 mm^2^ and the matrix size 64 × 64, yielding an in-plane isotropic spatial resolution of 3.125 mm. An automated high-order shimming method based on spiral acquisitions will be used before acquiring fMRI scans. All participants will undergo the task-free fMRI scan after being instructed only to remain awake with their eyes closed.

White matter diffusion tractography imaging (DTI) will be utilized to identify anatomical connections between functionally correlated regions. Anatomic T1-weighted scans will be obtained on a 3 T Siemens scanner with the following parameters: repetition time = 2100 ms; flip angle = 12°; slice thickness = 1.5 mm; inversion time = 1100 ms; matrix = 192 × 256; field of view = 172.5 mm; echo time = 3.87 ms. The DTI will be acquired in one non-weighted and six diffusion-weighted non-collinear directions with an echo-planner sequence with diffusion weighting 9b value of 1000 smm^−1^. A dual spin echo will be used to minimize distortion due to eddy currents. Imaging parameters are: repetition time = 6100 ms; flip angle = 90°; field of view = 178 × 219; matrix = 104 × 128; voxel size = 1.71 × 1.71. × 4 mm; number of averages = 4; echo time = 92 ms.

The control group will do baseline questionnaires and investigations similar to participants in the two intervention arms. They will do the neuroimaging at baseline and three months as well as the other investigations, similar to those in the two intervention arms. The only difference is the control group will not have blood samples taken at three months.

The fMRI scanning will be task-free. It is non-invasive and does not involve any injection of tracer dyes.

Caregivers will be asked to complete the Zarit Burden Scale at baseline, three months, and nine months. This is optional and caregivers can choose not to complete this scale and this will have no impact on subject participation. Caregivers will be asked to complete a portion of the Clinical Dementia Rating at baseline (if they are present or available) (Fig. 2).

### Recruitment

Participants will be recruited from amongst elderly individuals living around TaRA@JP and known to the research center. Only participants who agreed to be contacted for future research and known to have early cognitive impairment will be contacted by phone and invited to participate in the current study.

Others who have heard about the study will be recruited if they meet inclusion criteria and reside within close proximity to the research site (TaRA@JP) for travel arrangements.

They will be informed of the study and provided with a copy of the invitation letter and participant information sheet – both English and Mandarin versions will be made available to them. They will be given time to read the document in a private area at TaRA@JP and given time to consider whether to agree to participate in the study.

Nurses will interpret orally for those who are illiterate and for those who do not understand English. A Short Consent Form (Mandarin) will be used for the participant’s signature (or thumb print) and an impartial witness will be present and sign the document as well. If a translator is involved in the consent taking, he/she will also sign the consent form.

### Outcomes

Primary outcome measures:

1. Improvements in neuropsychological test scores at three months and nine months;
2. Positive changes in cerebral functioning at three months.

Secondary outcome measures:

1. Improvement in GDS and GAI scores at three months and nine months;
2. Increase in telomere lengths at three months and nine months.

### Sample size justification

To obtain the confidence interval with the width of 1 standard deviation (SD), which has a 90% chance to include the true difference of mean change in neuropsychological test score at three months between the intervention and control groups, requires 22 participants in each group. By considering the 25% drop-out rate, 30 participants will be required in each group. Thus, the total sample size will be 90 participants (30 in the AT intervention group, 30 in MRA, and 30 in the control group).

### Assessments and visit schedule

#### Pre-baseline

Screening, consent taking, and eligibility criteria are checked. Those with high GDS/GAI scores will go through a SCID examination to evaluate the presence of a major psychiatric disorder. This will be done by psychiatrists in the study team who have been trained to do this examination. Any participants found to have a psychiatric disorder through the SCID examination will be referred for follow-up by a medical professional. A standard referral letter has been prepared.

#### Baseline

Demography questionnaire, basic health screen, functional assessment, neuropsychological tests, neuroimaging, and blood investigations are carried out.

#### Three months

Basic health screen (excluding height), functional assessment, neuropsychological tests, neuroimaging, and blood investigations are repeated.

#### Nine months (study end)

Basic health screen (excluding height), functional assessment, neuropsychological tests, and blood investigations are repeated.

The time taken for the health screening, completion of the demographic data, main questionnaire, and blood sample collection will take about 1 h.

The neuropsychological tests will take about 1 h 45 min. The baseline visit to TaRA@JP will take about 3 h. Subsequent visits at three months and nine months will take 2 h.

The fMRI scans will be conducted at CIRC in NUS and the appointment will be on another day. The imaging sessions will take 1 h. However, the time within the scanner is about 45 min.

See Tables 1 and 2 for the visit schedules for the interventions and control groups, respectively.

**Table 1:** Visit schedule for intervention arms

Pre-baseline/Baseline	Three months	Nine months (study end)
Informed consent process	Main questionnaire	Main questionnaire
Eligibility screening	Neuropsychological tests	Neuropsychological tests
Demographic data	MRI scan	Clinical Dementia Rating
Main questionnaire	Weight, vital Signs	Weight, vital signs
Neuropsychological tests	Blood sample collection	Blood sample collection
Clinical Dementia Rating		
MRI scan		
Basic health screen		
Blood sample collection

**Table 2:** Visit schedule for controls

Pre-baseline/Baseline	Three months	Nine months (study end)
Informed consent process	Main questionnaire	Main questionnaire
Eligibility screening	Neuropsychological tests	Neuropsychological tests
Main questionnaire	MRI scan	Clinical Dementia Rating
Demographic data	Weight, vital signs	Weight, vital signs
Neuropsychological tests		Blood sample collection
Clinical Dementia Rating		
MRI scan		
Basic health screen		
Blood sample collection		

Blood samples (3 mL) will be collected at three time-points for those in the AT and MRA arms (baseline, three months, nine months) for telomere lengths. For the control group, it will be done twice, at baseline and at nine months.

Samples will be stored immediately in a mini-fridge (4 °C) at TaRA@JP and transported within 12 h of collection to the labs.

### Risks and safety monitoring

Risks include some pain and bruising during blood sampling. To minimize risks associated with the blood, sampling only one venipuncture will be performed at each of the three time-points and blood will be collected using vacutainers. Trained nurses will perform the procedure.

Some may experience some discomfort from the noise and the enclosed space within the scanner. Participants will be informed about the noise and ear plugs will be provided. If the noise is intolerable and the participant wishes to discontinue with the scanning, the scanning will be stopped.

### Data management

Data forms will be coded with a study number (starting with 001). Data will be entered and stored on a standalone computer and the information will be password protected. Only the principal investigator, investigators, and study coordinator in the project will have access to the data for analysis. In accordance with NUS data management policy (DPRT-2011-04), data will be kept for ten years after the research is completed and all data (electronic and hard copy) will be destroyed after the storage period.

The investigators and the trial coordinators will monitor that the informed consent process is conducted appropriately and that informed consent was obtained prior to proceeding with any study procedures. Only participants who meet study eligibility criteria will be enrolled.

Participants’ data will be identified only by a study number. Only the principal investigator will have access to identifiers that can link the data to the individual participant. De-identified data will be collected and analyzed as specified in the protocol. Participants who drop out will be noted and their reasons documented. Primary and secondary end-points will be reviewed to ensure data are correctly entered and meet protocol requirements.

### Statistical analyses

All efficacy analyses will be carried out on an intention-to-treat (ITT) basis. That is, all randomized participants will be included in the analysis and the intervention group of participants will be according to the randomization list planned prior to the intervention commencement.

The primary outcomes – neuropsychological test scores – at baseline, three months, and nine months will be modeled using the linear mixed model for repeated measurements. Differences in the estimated means of the changes at three and nine months from baseline between the intervention and control groups and their associated 90% and 95% confidence intervals will be calculated. Further, analysis may be performed to adjust the model for potential confounders, such as age, gender, and education level. Secondary outcomes (cerebral functioning, the GDS and GAI scores, and telomere lengths) will be analyzed similar to the primary outcomes.

Demographic and other baseline characteristics and reported adverse events and serious adverse events will be summarized using descriptive statistics.

## Discussion

Art and music activities have been described as “empowering tools that can assist in the aging process” [29]. Evidence suggests that training in these areas strengthen attention systems and improve cognition. However, the research to date is still limited on the effectiveness of incorporating these activities as part of care plans for the elderly with cognitive impairment. Furthermore, it is unlikely that these activities will “always improve general cognition” [30]. This study will more systematically determine whether both these activities will be of benefit for elderly with mild cognitive impairment and will provide additional psychosocial interventions that will be of use to this group.

### Trial status at the time of manuscript submission

The recruitment commenced in 13 June 2016 and the trial will end in April 2017.

## Additional file

Additional file 1: SPIRIT Checklist. Recommended items to address in a clinical trial protocol and related documents. (PDF 122 kb)

## Abbreviations

ADL: Activities of Daily Living
CDR: Clinical Dementia Rating
CTT: Color Trail Test
DSB: Digit Span Backward
DSF: Digit Span Forward
DTI: Diffusion tractography imaging
fMRI: Functional magnetic resonance imaging
GAI: Geriatric Anxiety Inventory
GDS: Geriatric Depression Scale
IADL: Instrumental Activities of Daily Living
MCI: Mild cognitive impairment
MMSE: Mini-Mental State Examination
PDQ: Perceived Deficits Questionnaire
RAVLT: Rey Auditory Verbal Learning Test
SCID: Structured Clinical Interview for DSM Disorder
TaRA@JP: Training and Research Academy at Jurong Point
